# Antioxidant Activity, Total Polyphenol Content, and Cytotoxicity of Various Types of Starch with the Addition of Different Polyphenols

**DOI:** 10.3390/molecules30112458

**Published:** 2025-06-04

**Authors:** Dominika Kwaśny, Barbara Borczak, Paweł Zagrodzki, Joanna Kapusta-Duch, Ewelina Prochownik, Ivo Doskočil

**Affiliations:** 1 Department of Human Nutrition and Dietetics, Faculty of Food Technology, University of Agriculture in Kraków, Aleja Mickiewicza 21, 31-120 Kraków, Poland; joanna.kapusta-duch@urk.edu.pl; 2 Department of Food Chemistry and Nutrition, Jagiellonian University Medical College, Medyczna 9, 30-688 Kraków, Poland; ewelina.gajdzik@uj.edu.pl (E.P.); pawel.zagrodzki@uj.edu.pl (P.Z.); 3 Department of Microbiology, Nutrition and Dietetics, Faculty of Agrobiology, Food and Natural Resources, Czech University of Life Sciences Prague, Kamýcká 129, 165 00 Prague, Czech Republic; doskocil@af.czu.cz

**Keywords:** antioxidant activity, total polyphenol content, cytotoxicity, starch, polyphenol, quercetin, (+)-catechin, epigallocatechin gallate, *trans*-ferulic acid

## Abstract

Given the high incidence of diet-related diseases, including type 2 diabetes and cancer, there is a growing need to explore new strategies for their prevention. Although polyphenols are known to reduce starch digestibility and lower the in vitro glycemic index, their antioxidant capacity and cytotoxic properties, when complexed with starches, remain underexplored. Therefore, this study aimed to investigate the antioxidant activity, total polyphenol content, and cytotoxic potential of polyphenol–starch complexes formed using common dietary polyphenols—(+)-catechin, epigallocatechin gallate, hesperidin, naringenin, *trans*-ferulic acid, *p*-coumaric acid, quercetin, and kaempferol—and widely consumed starches from wheat, rice, potato, and maize. Antioxidant activity (FRAP and DPPH) together with the total polyphenols content (Folin–Ciocalteu) were tested: (1) before (undigested) enzymatic hydrolysis of the tested sample; (2) after (digested) enzymatic hydrolysis of the tested sample and (3) after hydrolysis of the sample and its centrifugation (supernatant). Cytotoxicity against colon cancer (Caco-2, HT29) and normal colon (CCD 841CoN) cell lines were determined in vitro by the MTT method. In undigested samples, the highest antioxidant activity was obtained with the addition of quercetin to wheat, rice, and maize starch (6735.8 µmol Fe^2+^/g d.m., 678.8, 539.4 µmol Trolox/g d.m., respectively), and epigallocatechin gallate to wheat, rice, potato, and maize starch (692.1, 538.0, 625.8, 573.6 µmol Trolox/g d.m., respectively). In digested samples, the highest antioxidant activity was obtained with the addition of quercetin to wheat and rice starch (2104.5 µmol Fe^2+^/g d.m., 742.1 µmol Trolox/g d.m., respectively). In the case of the natant of the digested samples, the highest value was recorded for the addition of (+)-catechin to potato starch and *trans*-ferulic acid to maize starch (823.7 µmol Fe^2+^/g d.m., 245.1 µmol Trolox/g d.m., respectively). The addition of quercetin to wheat and rice starch and (+)-catechin to potato starch (0.239, 0.151, 0.085 g gallic acid/g d.m., respectively) resulted in the highest total polyphenol content. Furthermore, quercetin demonstrated the most significant level of cytotoxic activity against the tumor cell line Caco-2 (IC_50_ = 275.6 µg/mL; potato starch). Overall, quercetin was identified as the most significant or one of the most significant for all parameters evaluated.

## 1. Introduction

As shown in previous studies, the addition of polyphenols to starch influences starch digestibility and its in vitro glycemic index [1,2]. While the in vitro digestibility of starch is reduced by the addition of phenolic compounds, the antioxidant properties of the starch are concomitantly enhanced [3].

Starch is prevalent in various foods: whole grains (brown rice, oats, and whole wheat products like bread or pasta), legumes (lentils, beans, and chickpeas), tubers (potatoes, sweet potatoes, and yams), and refined products (white bread and pastries). Aside from starch content, whole grains are also rich in fiber and nutrients. Legumes, by contrast, are a source of resistant starch and protein. Tubers are a valuable source of vitamins and slowly digestible starch (SDS). Refined products contain rapidly digestible starches (RDS) that have been shown to cause sharp increases in blood sugar levels. The integration of whole grains and legumes, in conjunction with a restriction of refined starches, has been demonstrated to engender favorable health outcomes [4,5,6,7,8]. Therefore, as a complex carbohydrate, starch forms the basis of the diet, providing the greatest proportion of daily energy intake [9]. However, it has been established that the majority of starchy foods have a high glycemic index. Examples include white/whole wheat bread, white rice, boiled potatoes, and cornflakes [10,11].

Products with high glycemic index are rich sources of RDS [12]. It has been established that RDS is rapidly broken down and absorbed in the small intestine, leading to a swift rise in blood sugar levels. Rigorous research has proven that these pronounced fluctuations in blood glucose can impose a considerable burden on the body’s capacity to sustain glucose equilibrium. This stress may play a pivotal role in the onset of obesity, insulin resistance, and diabetes [13,14,15]. In contrast, SDS has been demonstrated to offer several health benefits, including improved glycemic control, enhanced diabetes management, augmented cognitive function, and enhanced appetite control. Research has demonstrated that, in comparison with RDS, SDS results in considerably more protracted and diminished fluctuations in blood glucose, insulin, and non-esterified fatty acid (NEFA) levels, thereby signifying a more stable metabolic response [16,17]. Furthermore, resistant starch (RS) that reaches the large intestine can serve as a nutrient source for gut microbes. During fermentation, the production of hydrogen, carbon dioxide, methane, and short-chain fatty acids occurs. These by-products have been demonstrated to support gastrointestinal health and assist in the regulation of blood sugar and insulin levels following meals, thereby conferring beneficial effects on overall health [18,19,20].

Phenols have been demonstrated to possess significant antioxidant properties, a factor that is particularly relevant when considering the potential consequences of excessive amounts of free radicals. Human performance has long been associated with physical and chemical interactions with the environment, which is now significantly degraded and polluted. The presence of pollutants in the environment has been proven to exert a detrimental effect on human antioxidant status through the production of reactive oxygen species (ROS). In addition to environmental pollution, there are other factors that can cause the formation of excessive amounts of free oxygen species in the body, including UV radiation from the sun, excessive physical exertion, an unhealthy diet, alcohol consumption, and smoking. These free radicals have been demonstrated to be capable of inflicting damage to cells and tissues, with the potential to result in the development of type 2 diabetes, cardiovascular diseases, and cancer, among other health complications. In order to counteract the harmful effects of ROS, it is important to increase dietary antioxidant intake, which helps strengthen the body’s defense mechanisms [21,22,23,24,25,26,27]. Therefore, the industrial utilization of polyphenols is predominantly concentrated within the domain of functional beverages (44%) and then functional foods (33%), chiefly attributable to the substantial demand for polyphenol-rich consumables [28].

The considerable versatility of polyphenols as agents that can improve human health is a significant reason why they remain among the most extensively researched bioactive food compounds. However, research on the beneficial effects of polyphenols frequently neglects to address concerns regarding their potential risks. The field of toxicity studies on dietary polyphenols remains underdeveloped, and the extent of knowledge about their acute or chronic toxic effects is still limited. Despite the prevalent notion that plant-based compounds are inherently safe, comprehensive safety data are lacking. A significant number of food and feed products containing polyphenols undergo minimal toxicity testing, which could lead to harmful effects. Higher doses of polyphenols, especially when consumed in functional foods or pharmaceutical products, may have deleterious effects on consumers of different ages, sexes, and health conditions [28,29].

A substantial body of research has been conducted on the antioxidant activity of polyphenols, particularly within the same polyphenol groups (flavones, flavanols, flavonols, phenolic acids, etc.). In contrast, there is a notable lack of research on the antioxidant activity of polyphenol-starch complexes. Moreover, the existing research is largely confined to the study of specific individual starches and polyphenols [13,14,15,16,17,18,19,20,21,22]. Furthermore, these studies differ significantly in their methodologies. Consequently, a comparison of these results is not feasible. The present study aims to address this knowledge gap by comparing the antioxidant activity of polyphenol-starch complexes with a focus on common polyphenols found in everyday foods and the most widely consumed starches across diverse geographical regions.

Therefore, the objective of the present study was twofold: first, to investigate the antioxidant activity and total polyphenol content of the polyphenol-starch samples that exhibited the greatest efficacy in reducing starch digestibility and in vitro glycemic index, and second, to assess the cytotoxic potential of the samples.

## 2. Results and Discussion

### 2.1. Antioxidant Activity

Polyphenol-starch complexes were subjected to testing in three variants: (1) prior to starch digestion (the reaction mixture without amylolytic enzymes), (2) following digestion (the reaction mixture containing hydrolysis products of polyphenol-starch complexes), and (3) following digestion, using only the supernatant (the soluble fraction of the reaction mixture containing hydrolysis products of polyphenol-starch complexes).

The highest value of antioxidant activity measured by the FRAP method was observed for quercetin added to wheat starch for both digested and undigested samples (Table 1). The values were 2104.5 and 6735.8 µmol Fe^2+^/g dry matter per 1 mg polyphenol, respectively (*p* < 0.05). In the case of the supernatant of the digested samples, the highest value was recorded for the addition of (+)-catechin to potato starch (823.7 µmol Fe^2+^/g dry matter per 1 mg polyphenol, *p* < 0.05, Table 1).

In the case of the DPPH· method, the highest results were observed for the addition of quercetin to rice and maize starches and for epigallocatechin gallate across all tested starches (Table 2). The values were 678.8, 539.4, 692.1, 538.0, 625.8, and 573.6 µmol Trolox/g dry matter per 1 mg polyphenol, respectively (*p* < 0.05). Conversely, for samples that had undergone digestion, the highest result was recorded for quercetin added to rice starch (742.1 µmol Trolox/g dry matter per 1 mg polyphenol, *p* < 0.05). In the case of the supernatant of the digested samples, the highest value was obtained for the addition of *trans*-ferulic acid to maize starch (245.1 µmol Trolox/g dry matter per 1 mg polyphenol, *p* < 0.05, Table 2).

For the samples that had undergone digestion (supernatant), antioxidant activity measured by the FRAP and DPPH· methods was significantly higher than that of the control sample only in the samples containing (+)-catechin, epigallocatechin gallate, and *trans*-ferulic acid. This may be attributed to the greater water solubility of these polyphenols compared to the others tested [30,31,32,33,34,35,36,37].

The antioxidant action of polysaccharides depends on multiple factors, including their solubility, sugar ring structure, molecular weight, the presence of positive or negative charges, covalently bound phenolic compounds, and protein moieties [38].

In this study, the antioxidant activity, as determined by both the FRAP and DPPH· methods, was below the limit of quantification (LOQ) for wheat, rice, potato, and maize starch in the absence of added polyphenols (Table 1 and Table 2).

The chemical structure of polyphenols studied in the context of starch digestibility inhibition and formation of polyphenol-starch complexes has been extensively described in our previous publications [1,2]. The inhibitory effect of polyphenols on starch digestibility and their capacity to form starch–polyphenol complexes are closely related to their chemical structure. These indigestible complexes might be classified as a new fraction of dietary fiber not previously described in the literature and may be referred to as novel resistant starch type 6 (RS6), in addition to the already known types RS1-RS5. Key structural elements—such as the number and position of hydroxyl groups, the presence of a galloyl moiety, conjugated double bonds, and the absence of glycosylation or methylation—play a critical role in determining the strength of interactions with digestive enzymes and starch molecules. Epigallocatechin gallate exhibited the strongest inhibitory activity due to its high hydroxyl content, unique galloyl group, and conjugated system, which enable robust hydrogen bonding and hydrophobic interactions with α-amylase and starch granules. Other flavonoids, such as quercetin and (+)-catechin, also demonstrated potent inhibitory effects, although their efficiency varied with dose and starch type. Flavonols, flavanones, and phenolic acids showed moderate to low activity, influenced by their simpler structures and lower affinity for enzyme-active sites. Overall, polyphenol–starch interaction efficacy is structure-dependent, with specific configurations enhancing resistance to enzymatic hydrolysis and promoting the formation of non-digestible complexes [1,2].

The antioxidant activity of polyphenols is influenced by both the number and position of hydroxyl groups on the aromatic ring, as well as the nature of the substituent present [21]. An increase in the number of hydroxyl groups is associated with enhanced antioxidant activity [39,40].

Flavonoids containing a catechol group in the B ring (such as quercetin and (+)-catechin) exhibit enhanced antioxidant activity. In addition to the catechol grouping, quercetin has a double bond between carbons C2 and C3, the 3-OH group, and the 4-oxo function, all of which further contribute to its antioxidant properties [41,42]. This may explain the observation that quercetin, when used as an additive to diverse starch types, resulted in a notably elevated antioxidant activity. Conjugation of starch with quercetin has been demonstrated to significantly enhance the antioxidant activity of starch in other studies. According to Cirillo et al., the antioxidant capacity (DPPH· radical scavenging activity) of the starch-quercetin conjugate was significantly higher than the control sample [43]. The study conducted by Liu et al. also demonstrated that the quercetin-starch conjugate exhibited markedly higher DPPH· radical scavenging activity compared to *C. auriculatum* starch [44]. Similar findings were reported by Xia et al., whose studies focused on a carboxymethyl sweet potato starch-quercetin conjugate [45].

The structure formula of (+)-catechin also includes, like quercetin, the 3-OH group in the C ring. However, it is known that the 3-OH, when considered in conjunction with the adjacent double bond in the C ring, has a critical influence on antioxidant activity. The elimination of either structural feature results in a concomitant loss of this effect [42]. The results obtained for starch combined with (+)-catechin were significantly lower compared with those for quercetin in most cases except for undigested potato starch in both the FRAP and DPPH· methods and for all samples when antioxidant activity was measured in the supernatant. Hu et al. also demonstrated that the conjugation of starch with (+)-catechin significantly enhances the antioxidant activity of starch [46].

Similarly, for kaempferol, although its structure formula differs from that of quercetin only in terms of the number of hydroxyl groups in the B ring, the presence of a single hydroxyl group in the B ring appears to have a minimal impact on the antioxidant potential of kaempferol, even when considered in conjunction with the conjugated double bond system and the 3-hydroxyl group [42]. The results for starch with kaempferol were generally significantly lower than those obtained with quercetin-with the exception of undigested rice and potato starch, digested potato starch (FRAP method), and undigested wheat starch (DPPH· method).

Of the flavonoids examined (quercetin, kaempferol, (+)-catechin, epigallocatechin gallate, hesperidin, and naringenin), in addition to quercetin and (+)-catechin, epigallocatechin gallate also contains a catechol group in its structure. Furthermore, it contains a 3-OH group in the B ring. However, the presence of the 3-OH group does not appear to enhance antioxidant activity [42]. In addition, epigallocatechin gallate lacks a double bond between atoms C2 and C3, a 3-OH group in the C ring, and a 4-oxo function. The results for starch combined with epigallocatechin gallate were significantly lower in all cases compared to those with quercetin, and in some cases also lower than those with kaempferol–specifically for digested and undigested rice starch, digested wheat starch, undigested maize starch (FRAP method), and undigested wheat starch (DPPH· method). A study conducted by Yong et al. has demonstrated that the conjugation of epigallocatechin gallate with dialdehyde starch significantly increases the antioxidant activity of starch [47].

In the case of hesperidin and naringenin, the catechol group in the B ring, the double bond between the C2 and C3 atoms, and the 3-OH group in the C ring are all present in their respective structures. Conversely, a 4-oxo function is present, yet its effect on antioxidant activity is observed only when it co-exists with the C2-C3 double bond [42]. The present study found no significant differences in the antioxidant activity of samples containing hesperidin and naringenin. However, other studies have reported higher antioxidant activity for both flavanones compared to quercetin [42,48].

As with flavonoids, the antioxidant activity of phenolic acids also depends on the number of hydroxyl groups in the molecule. Both *trans*-ferulic and *p*-coumaric acid contain a single hydroxyl group. The structure of *trans*-ferulic acid includes an additional methoxyl group, which is known to enhance its antioxidant activity [42,48,49]. In this study, the antioxidant activity of starch combined with *trans*-ferulic acid was also significantly higher than that of starch with *p*-coumaric acid in all undigested starch samples and most digested samples–with the exception of rice starch (FRAP method).

### 2.2. Total Phenolic Content (TPC)

In the analysis of total polyphenol content, the highest value for undigested samples was observed for quercetin added to wheat starch (0.239 g gallic acid/g dry matter per 1 mg polyphenol, *p* < 0.05, Table 3), consistent with the antioxidant activity measured by the FRAP method. Conversely, for digested samples, the highest value was recorded for quercetin added to rice starch (0.151 g gallic acid/g dry matter per 1 mg polyphenol, *p* < 0.05), in agreement with the antioxidant activity measured by the DPPH· method. For digested samples measured in the supernatant, the highest value was obtained for (+)-catechin added to potato starch (0.085 g gallic acid/g dry matter per 1 mg polyphenol, *p* < 0.05, Table 3).

In this study, the total polyphenol content in wheat, rice, potato, and maize starch without the addition of polyphenols was below the limit of quantification (LOQ) (Table 3).

For the digested (supernatant) samples, a significantly higher level than the control was observed only in the samples containing (+)-catechin and epigallocatechin gallate.

In the study conducted by Deng et al., the polyphenol content of debranched rice starch-polyphenol inclusion complexes was investigated. Among the compounds studied were quercetin-starch and ferulic acid-starch complexes. The reported values were 12.77 and 14.21 mmol/g starch, respectively (*p* < 0.05) [50]. In the present study, differences between these two polyphenols were observed only in the digested samples. The TPC values for rice starch-quercetin and rice starch-*trans* ferulic acid conjugates were 0.151 and 0.043 g gallic acid/g dry matter per 1 mg polyphenol, respectively (*p* < 0.05).

The potential value of TPC is attributable to the stability of the polyphenol-starch complexes. This stability is influenced by a number of factors, including molecular weight and the number of hydroxyl groups present in the polyphenol’s structure [51]. The polyphenol-starch complexes were divided into inclusion and non-inclusion complexes. In inclusion complexes, the polyphenol enters the helical cavity of the starch, while in non-inclusion complexes, it is trapped between helices [52,53,54]. It appears that inclusion complexes are more stable. Polyphenols with a lower molecular weight have a greater capacity to form such complexes. Conversely, polyphenols with a higher molecular weight tend to reduce complex stability, which can lead to starch aggregation [51,55]. The molecular weight values of the tested polyphenols–(+)-catechin, epigallocatechin gallate, hesperidin, naringenin, *trans*-ferulic acid, *p*-coumaric acid, quercetin, and kaempferol–are 290.27, 458.37, 610.56, 272.25, 194.18, 164.16, 302.24, 286.24, respectively [56].

The stability of polyphenol-starch complexes is also influenced by the number of hydroxyl groups present in the polyphenol moiety. The presence of hydroxyl groups is crucial for facilitating the formation of hydrogen bonds between the polyphenol and starch. It has been established that an increase in the number of hydroxyl groups results in greater stability of the complex [51,57,58]. Among the polyphenols examined in this study, epigallocatechin gallate was found to possess the highest number of hydroxyl groups per molecule. However, it did not exhibit the highest TPC value, which can be explained by the relatively high molecular weight of this compound. It has been established that (+)-catechin and quercetin possess fewer hydroxyl groups compared with epigallocatechin gallate. However, their molecular weights are significantly lower, thereby enabling them to form stable complexes with starch. Consequently, this phenomenon accounts for their elevated TPC values. The remaining polyphenols (i.e., kaempferol, hesperidin, naringenin, *trans*-ferulic acid, and *p*-coumaric acid) possess a lower number of hydroxyl groups. The phenolic acids that were examined in this study have the lowest molecular weights, yet they do not yield the highest TPC results. This outcome may be attributed to the presence of only a single hydroxyl group in each molecule of these acids.

### 2.3. Cytotoxicity

The cytotoxicity of the polyphenol-starch complexes was investigated against the colon cancer cell lines Caco-2 and HT29 and the normal colon cell line CCD 841CoN in order to evaluate the potential detrimental effects of the tested polyphenols at the applied doses on both cancerous and normal cells. Caco-2 is a human epithelial cell line that was originally derived from a human colon carcinoma, while the HT29 cell line is derived from a human colon adenocarcinoma [59,60]. CCD 841CoN is a human colonic epithelial cell line [61]. In this study, it was demonstrated that potato starch, in conjunction with quercetin, exhibited the highest degree of toxicity toward the Caco-2 cancer cell line (IC_50_ = 275.6 µg/mL, *p* < 0.05). A significantly higher IC_50_ value was reported for the same sample against the HT29 cancer cell line (IC_50_ = 470.5 µg/mL, *p* < 0.05). Toxicity values for the remaining samples exceeded the upper limit of the LOQ for both the Caco-2 and HT29 cell lines. In the case of the normal cell line CCD 841CoN, all tested polyphenol-starch complexes exhibited toxicity values above the LOQ. Therefore, none of the tested samples demonstrated toxicity toward this cell line (Table 4).

There have been studies conducted on the toxicity of polyphenols, focusing on cancer cell lines, including Caco-2 and HT29 [62,63,64,65,66,67,68,69,70,71]. Volstatova et al. reported greater quercetin toxicity against the Caco-2 line than against HT29-MTX cells [62]. According to Agullo et al., quercetin was found to be more toxic to HT29 cells than kaempferol [67]. Similar results were obtained by Kuntz et al., who showed that quercetin was more toxic than kaempferol to both HT29 and Caco-2 cancer cells [64].

This study is novel in that it does not examine the cytotoxicity of individual polyphenols but rather focuses on the cytotoxicity of starch–polyphenol complexes–an area that, to the best of our knowledge, has not been previously investigated in the literature.

## 3. Materials and Methods

### 3.1. Sample Preparation

#### 3.1.1. Preparation of Starch–Phenolic Complexes

5% *w*/*v* gels of wheat, rice, potato, and maize starch (Sigma Aldrich, S5127, St. Louis, MO, USA; Sigma Aldrich, S7260, St. Louis, MO, USA; Chempur, Piekary Śląskie, Poland; Biomus, Lublin, Poland, respectively) were prepared. In addition to starch, two polyphenols from four different groups were selected: (+)catechin hydrate (Sigma Aldrich 22110, St. Louis, MO, USA) and epigallocatechin gallate (Sigma Aldrich PHR1333, St. Louis, MO, USA) (flavonols), quercetin (Sigma Aldrich Q4951, St. Louis, MO, USA) and kaempferol (Sigma Aldrich K0133, St. Louis, MO, USA) (flavonols), naringenin (Sigma Aldrich N5893, St. Louis, MO, USA) and hesperidin (Sigma Aldrich H5254, St. Louis, MO, USA) (flavanones), and *trans*-ferulic acid (Sigma Aldrich 128708, St. Louis, MO, USA) and *p*-coumaric acid (Sigma Aldrich C9008, St. Louis, MO, USA) (phenolic acids). Each of the compounds mentioned above was added separately to the starch before (first, the addition of polyphenol to the starch took place, then the gelatinization of the starch at 90 °C, and finally cooling of the samples to 37 °C), and after making gels (first the gelatinization of the starch in 90 °C took place, then cooling of the samples to 37 °C, and finally the addition of polyphenol to the starch), in the following doses: 5, 10, and 20 mg [2].

#### 3.1.2. Selection of Samples for the Present Study

Samples that demonstrated optimal outcomes with regard to starch digestibility and estimated glycemic index [2] were selected and frozen until subsequent analyses (Table 5).

#### 3.1.3. Samples in Total (Before and After the Digestion Process)

The samples were thawed at room temperature and then placed in a laboratory shaker (BIOSAN Vortex TS-100, Warren, MI, USA). The samples were shaken for approximately 1 h. From each sample, 100 µL was taken and dissolved in 900 µL dimethyl sulfoxide (DMSO) (Chempur, Piekary Śląskie, Poland).

#### 3.1.4. Sample’s Supernatant (After the Digestion Process)

The samples were thawed at room temperature and then placed in a laboratory shaker (BIOSAN Vortex TS-100, Warren, MI, USA). The samples were shaken for approximately 1 h. The samples were then centrifuged (MPW MED. INSTRUMENTS 351R, Warszawa, Poland) at 1380 G for 10 min at 25 °C. The resulting supernatant was transferred to a 1500 µL Eppendorf tube. From each sample, 100 µL was taken and dissolved in 900 µL of DMSO reagent (Chempur, Piekary Śląskie, Poland).

### 3.2. Measurement of the Antioxidant Activity

#### 3.2.1. FRAP Method

The measurement of antioxidant activity using the FRAP (Ferric Reducing Antioxidant Potential) method was performed according to Benzie and Strain’s method [72], with some modifications. The FRAP reagent was prepared in a 2:1:1 ratio by mixing 0.3 mol/L acetate buffer (pH 3.6), TPTZ, and iron (III) chloride (20 mmol/L FeCl_3_·6H_2_O (Honeywell Fluka™ 44944, Charlotte, NC, USA)). Then 0.4 mL of acetate buffer, 0.05 mL of the sample tested, and 0.2 mL of the FRAP mixture were dispensed onto a 48-well plate. The prepared solution was then incubated at 37 °C for 30 min. Finally, its absorbance was measured at a wavelength of 593 nm using a microplate spectrophotometer (Synergy 2, BioTek, Winooski, VT, USA). The measurement required calibration against iron (II) ions. A standard calibration solution of 1.5 mmol/L ferrous sulfate (FeSO_4_·7H_2_O) (POCH, Gliwice, Poland)) was used (range of the calibration curve: 0 ÷ 1800 µmol Fe^2+^/L; LOQ = 65 µmol Fe^2+^/L).

#### 3.2.2. DPPH· Method

The determination of antioxidant activity using the synthetic DPPH· radical (1,1-diphenyl-2-picrylhydrazyl) was performed according to the Brand-Williams et al. method [73], with some modifications. To the 96-well plates, 88 µL of acetate buffer and 32 µL of the tested sample combined with DMSO (Chempur, Piekary Śląskie, Poland) were dispensed (according to the scheme: 4 µL of sample + 28 µL of DMSO, 8 µL of sample + 24 µL of DMSO, 16 µL of sample + 16 µL of DMSO, 32 µL of sample + 0 µL of DMSO), along with DPPH·. The mixture was then incubated at 37 °C for 30 min. Absorbance was measured at a wavelength of 515 nm using a plate reader (Synergy 2, BioTek, Winooski, VT, USA). The measurement of antioxidant activity required calibration against Trolox. Trolox solution was used as the standard calibration solution (range of the calibration curve: 0 ÷ 50 µmol Trolox/L; LOQ = 0.8 µmol Trolox/L).

### 3.3. Measurement of the Total Polyphenol Content (TPC)

The measurement of total polyphenol content was performed using the Folin–Ciocalteu reagent, according to Swain and Hillis [74], with some modifications. The Folin–Ciocalteu reagent (Sigma Aldrich 47641, St. Louis, MO, USA) was prepared by mixing it with distilled water in a 1:2 ratio. To a 48-well microplate, 540 µL of distilled water, 60 µL of the sample tested, 60 µL of a 7% sodium carbonate solution, and 30 µL of the Folin–Ciocalteu reagent solution were dispensed. The mixture was then incubated at 25 °C for 30 min. Absorbance was measured at a wavelength of 760 nm using a microplate spectrophotometer (Synergy 2, BioTek, Winooski, VT, USA). The measurement required calibration against gallic acid. A standard calibration solution of 0.3 g/L gallic acid was used (range of the calibration curve: 0 ÷ 0.35 g gallic acid/L; LOQ = 0.03 g gallic acid/L).

### 3.4. Measurement of the Cytotoxicity

Human colon cancer cells line Caco-2 and HT29, as well as colon normal cells line CCD 841CoN (ATCC, Rockville, MD, USA) were cultured in EMEM supplemented with 10% fetal bovine serum, 1% sodium bicarbonate, 1% sodium pyruvate, 5 mmol/L glutamine, 1% MEM non-essential amino acids, and 1% penicillin-streptomycin solution (10,000 units/mL of penicillin and 10 mg/mL of streptomycin). Cultures were incubated at 37 °C with 5% CO_2_, and media were replenished every 2–3 days, with passaging after every 7 days. Cell viability was measured using the modified MTT cytotoxicity assay developed by Mosmann [75]. Briefly, cells were seeded in 96-well plates at a density of 2.5 × 103 (Caco-2, HT29, and CCD 841CoN). After 24 h, the cells were treated with two-fold serial diluted samples (20% of digestate) for 72 h. Then, MTT reagent (1 mg/mL) in EMEM was added to each well and incubated for an additional 2 h at 37 °C with 5% CO_2_. Medium with MTT was removed, and the intracellular formazan product was dissolved in 100 μL of DMSO (Chempur, Piekary Śląskie, Poland). The absorbance was measured at 555 nm using a Tecan Infinite M200 spectrometer (Tecan Group, Männedorf, Switzerland), and the percentage of viability (IC_50_ value) was calculated when compared to an untreated control.

### 3.5. Data Analyses

The results obtained were presented as ranges of a minimum of three parallel repetitions, with the standard deviation around the mean. Multivariate analysis of variance (MANOVA) was utilized for the assessment of the impact of various polyphenolic compounds on the antioxidant activity, the total content of polyphenols, and the cytotoxicity of different types of starch. The significance of the observed differences was then determined using the Duncan test at a significance level of *p* < 0.05. All calculations were performed using Statistica v.13 software (Statsoft, Inc., Tulsa, OK, USA).

## 4. Conclusions

It is imperative to acknowledge the significance of studies in this field, as they serve to evaluate the antioxidant and cytotoxic properties of starch samples that have been complexed with polyphenols. These complexes have already been shown to reduce the digestibility of various starch types, thereby lowering their glycemic index. The results may support the development of traditional starchy products with enhanced functional properties such as lower glycemic index, increased antioxidant activity, and cytotoxicity potential against cancer cells without altering organoleptic characteristics. The present study demonstrates that the addition of selected polyphenols significantly enhances the antioxidant activity and total polyphenol content of the tested starches (*p* < 0.05). The highest values were obtained with the addition of quercetin, epigallocatechin gallate, (+)-catechin, and *trans*-ferulic acid to the starches (*p* < 0.05). Furthermore, the addition of quercetin to potato starch had a significant effect on the viability of cancer cell lines Caco-2 and HT29 (*p* < 0.05). Follow-up studies on samples with the best results in terms of starch digestibility and estimated glycemic index are necessary to assess physicochemical properties, such as amylose-to-amylopectin ratio and morphological changes in starch granules resulting from polyphenol addition. Moreover, the next phase of research will involve investigating the starch digestion inhibition and antioxidant and cytotoxic potential of starch-rich products, such as bread. In addition, future studies could include in vivo measurement of the glycemic index in human volunteers following the consumption of starch-rich products enriched with specific polyphenols.

## Figures and Tables

**Table 1:** The antioxidant activity measured by the FRAP method [µmol Fe^2+^/g dry matter per 1 mg polyphenol ***].

Sample			Digestion	
Starch	Polyphenol	Before	After	After (Natant)
Wheat	-	<LOQ ^a^*	<LOQ ^a^	<LOQ ^a^
	(+)-catechin	493.7 ± 9.3 ^c,d,e,f^	587.1 ± 17.8 ^d,e,f^	528.1 ± 18.0 ^d,e^
	Epigallocatechin gallate	1123.0 ± 4.6 ^h,i,j^	359.0 ± 10.2 ^a,b,c,d^	323.0 ± 19.9 ^b^
	Naringenin	<LOQ ^a^	<LOQ ^a^	<LOQ ^a^
	Quercetin	6735.8 ± 8.0 ^m^**	**2104.5 ± 8.8 ^h^**	<LOQ ^a^
	Kaempferol	931.4 ± 13.0 ^g,h,i^	812.7 ± 2.8 ^e,f^	<LOQ ^a^
Rice	-	<LOQ ^a^	<LOQ ^a^	<LOQ ^a^
	Epigallocatechin gallate	866.9 ± 1.2 ^f,g,h^	532.0 ± 8.7 ^b,c,d,e^	355.9 ± 34.8 ^b,c^
	Hesperidin	142.9 ± 1.3 ^a,b,c^	145.0 ± 48.4 ^a,b,c^	<LOQ ^a^
	Naringenin	<LOQ ^a^	<LOQ ^a^	<LOQ ^a^
	*Trans*-ferulic acid	571.6 ± 14.2 ^d,e,f,g^	373.9 ± 9.6 ^a,b,c,d^	671.8 ± 2.6 ^f^
	*p*-coumaric acid	135.3 ± 2.3 ^a,b,c^	135.7 ± 27.0 ^a,b^	127.3 ± 6.2 ^a^
	Quercetin	1420.5 ± 6.1 ^j^	1318.7 ± 44.1 ^g^	<LOQ ^a^
	Kaempferol	1416.2 ± 2.6 ^j^	950.7 ± 4.1 ^f^	<LOQ ^a^
Potato	-	<LOQ ^a^	<LOQ ^a^	<LOQ ^a^
	(+)-catechin	459.7 ± 5.2 ^b,c,d,e^	803.5 ± 4.8 ^e,f^	**823.7 ± 1.5 ^g^**
	Epigallocatechin gallate	746.7 ± 27.1 ^e,f,g,h^	545.4 ± 14.8 ^c,d,e^	459.0 ± 18.8 ^c,d^
	Hesperidin	63.2 ±22.1 ^a,b^	152.5 ±4.8 ^a,b,c^	<LOQ ^a^
	Naringenin	<LOQ ^a^	<LOQ ^a^	<LOQ ^a^
	*p*-coumaric acid	141.6 ± 0.2 ^a,b,c^	141.8 ± 7.9 ^a,b^	129.9 ± 10.1 ^a^
	Quercetin	183.5 ± 33.9 ^a,b,c,d^	968.0 ± 11.3 ^f^	<LOQ ^a^
	Kaempferol	38.4 ± 11.5 ^a^	730.8 ± 5.6 ^d,e,f^	<LOQ ^a^
Maize	-	<LOQ ^a^	<LOQ ^a^	<LOQ ^a^
	(+)-catechin	425.8 ± 7.2 ^b,c,d,e^	851.4 ± 5.2 ^e,f^	686.6 ± 20.5 ^f^
	Epigallocatechin gallate	1268.0 ± 5.3 ^i,j^	330.2 ± 26.2 ^a,b,c,d^	314.8 ± 26.6 ^b^
	*Trans*-ferulic acid	614.1 ± 4.5 ^e,f,g^	596.8 ± 6.2 ^d,e,f^	576.3 ± 27.0 ^e,f^
	Quercetin	3252.2 ± 3.8 ^l^	1335.7 ± 1.3 ^g^	<LOQ ^a^
	Kaempferol	2222.5 ± 0.8 ^k^	497.9 ± 4.1 ^b,c,d,e^	<LOQ ^a^

* The results are presented as means (±) relative standard deviation (RSD). Values marked with different letters in columns differ significantly at *p* < 0.05. The limit of quantification (LOQ) < 31.3 µmol Fe^2+^/g dry matter. ** Values in bold are the highest values within columns. *** The dry matter content of the tested samples was 4.54 ± 0.1% (wheat starch), 5.07 ± 0.2% (rice starch), 4.96 ± 0.4% (potato starch), 4.88 ± 0.2% (maize starch).

**Table 2:** The antioxidant activity measured by the DPPH· method [µmol Trolox/g dry matter per 1 mg polyphenol ***].

Sample			Digestion	
Starch	Polyphenol	Before	After	After (Natant)
Wheat	-	<LOQ ^a^*	<LOQ ^a^	<LOQ ^a^
	(+)-catechin	138.6 ± 23.1 ^a,b^	195.7 ± 12.4 ^b,c^	154.4 ± 2.5 ^b^
	Epigallocatechin gallate	692.1 ± 7.2 ^d^**	261.8 ± 8.7 ^c^	222.6 ± 5.5 ^c,d^
	Naringenin	<LOQ ^a^	<LOQ ^a^	<LOQ ^a^
	Quercetin	376.6 ± 21.6 ^c^	545.1 ± 1.8 ^e^	<LOQ ^a^
	Kaempferol	233.5 ± 14.3 ^b,c^	209.1 ± 11.9 ^b,c^	<LOQ ^a^
Rice	-	<LOQ ^a^	<LOQ ^a^	<LOQ ^a^
	Epigallocatechin gallate	**538.0 ± 30.2 ^d^**	270.9 ± 10.4 ^c^	206.5 ± 8.1 ^b,c,d^
	Hesperidin	<LOQ ^a^	34.3 ± 38.3 ^a^	<LOQ ^a^
	Naringenin	<LOQ ^a^	<LOQ ^a^	<LOQ ^a^
	*Trans*-ferulic acid	288.6 ± 10.1 ^b,c^	149.8 ± 58.7 ^b^	165.2 ± 52.8 ^b,c^
	*p*-coumaric acid	<LOQ ^a^	<LOQ ^a^	<LOQ ^a^
	Quercetin	**678.8 ± 0.1 ^d^**	**742.1 ± 14.7 ^f^**	<LOQ ^a^
	Kaempferol	228.7 ± 35.5 ^b,c^	201.4 ± 8.8 ^b,c^	<LOQ ^a^
Potato	-	<LOQ ^a^	<LOQ ^a^	<LOQ ^a^
	(+)-catechin	341.3 ± 24.5 ^c^	<LOQ ^a^	153.9 ± 24.2 ^b^
	Epigallocatechin gallate	**625.8 ± 3.2 ^d^**	<LOQ ^a^	166.4 ± 21.1 ^b,c^
	Hesperidin	<LOQ ^a^	40.8 ± 5.4 ^a^	<LOQ ^a^
	Naringenin	<LOQ ^a^	<LOQ ^a^	<LOQ ^a^
	*p*-coumaric acid	<LOQ ^a^	<LOQ ^a^	<LOQ ^a^
	Quercetin	381.7 ± 16.0 ^c^	438.9 ± 1.7 ^d^	<LOQ ^a^
	Kaempferol	150.0 ± 22.9 ^a,b^	<LOQ ^a^	<LOQ ^a^
Maize	-	<LOQ ^a^	<LOQ ^a^	<LOQ ^a^
	(+)-catechin	146.3 ± 26.8 ^a,b^	242.0 ± 67.3 ^b,c^	211.0 ± 0.0 ^b,c,d^
	Epigallocatechin gallate	**573.6 ± 47.3 ^d^**	283.0 ± 16.1 ^c^	159.3 ± 18.0 ^b,c^
	*Trans*-ferulic acid	283.7 ± 2.7 ^b,c^	294.3 ± 3.4 ^c^	**245.1 ± 3.7 ^d^**
	Quercetin	**539.4 ± 8.6 ^d^**	619.3 ± 0.7 ^e^	<LOQ ^a^
	Kaempferol	184.8 ± 18.1 ^a,b^	153.5 ± 16.4 ^b^	<LOQ ^a^

* The results are presented as means (±) relative standard deviation (RSD). Values marked with different letters in columns differ significantly at *p* < 0.05. The limit of quantification (LOQ) < 32.9 µmol Trolox/g dry matter. ** Values in bold indicate the highest values within columns. *** The dry matter content of the tested samples was 4.54 ± 0.1% (wheat starch), 5.07 ± 0.2% (rice starch), 4.96 ± 0.4% (potato starch), 4.88 ± 0.2% (maize starch).

**Table 3:** The total content of phenolic compounds (TPC) was measured by the Folin–Ciocalteu reagent method [g gallic acid/g dry matter per 1 mg polyphenol ***].

Sample			Digestion	
Starch	Polyphenol	Before	After	After (Natant)
Wheat	-	<LOQ ^a^*	<LOQ ^a^	<LOQ ^a^
	(+)-catechin	0.025 ± 11.6 ^a^	0.036 ± 3.8 ^a,b,c,d^	0.058 ± 64.0 ^b,c^
	Epigallocatechin gallate	0.069 ± 16.3 ^a,b,c,d,e,f,g^	0.050 ± 8.2 ^a,b,c,d,e,f^	0.068 ± 24.7 ^c,d^
	Naringenin	0.089 ± 21.8 ^d,e,f,g,h^	0.027 ± 7.4 ^a,b,c^	<LOQ ^a^
	Quercetin	0.239 ± 52.8 ^i^**	0.103 ± 1.9 ^g,h^	<LOQ ^a^
	Kaempferol	0.072 ± 16.1 ^a,b,c,d,e,f,g^	0.049 ± 9.3 ^a,b,c,d,e^	<LOQ ^a^
Rice	-	<LOQ ^a^	<LOQ ^a^	<LOQ ^a^
	Epigallocatechin gallate	0.036 ± 14.2 ^a,b,c^	0.039 ± 23.3 ^a,b,c,d^	0.042 ± 37.1 ^a,b,c^
	Hesperidin	0.030 ± 8.9 ^a,b^	0.032 ± 53.7 ^a,b,c,d^	<LOQ ^a^
	Naringenin	0.130 ± 8.2 ^h^	0.056 ± 2.3 ^c,d,e,f^	<LOQ ^a^
	*Trans*-ferulic acid	0.091 ± 10.2 ^d,e,f,g,h^	0.043 ± 56.3 ^a,b,c,d,e^	0.049 ± 49.4 ^a,b,c^
	*p*-coumaric acid	0.089 ± 0.4 ^d,e,f,g,h^	0.047 ± 50.2 ^a,b,c,d,e^	<LOQ ^a^
	Quercetin	0.124 ± 14.4 ^h^	**0.151 ± 19.3 ^i^**	<LOQ ^a^
	Kaempferol	0.104 ± 27.0 ^e,f,g,h^	0.061 ± 4.7 ^d,e,f^	<LOQ ^a^
Potato	-	<LOQ ^a^	<LOQ ^a^	<LOQ ^a^
	(+)-catechin	0.098 ± 22.8 ^e,f,g,h^	0.083 ± 23.2 ^f,g,h^	**0.085 ± 62.5 ^d^**
	Epigallocatechin gallate	0.053 ± 13.7 ^a,b,c,d,e^	0.049 ± 23.9 ^a,b,c,d,e^	0.036 ± 46.4 ^a,b^
	Hesperidin	0.022 ± 4.6 ^a^	0.027 ± 13.0 ^a,b,c^	<LOQ ^a^
	Naringenin	0.119 ± 3.2 ^g,h^	0.093 ± 13.7 ^g,h^	<LOQ ^a^
	*p*-coumaric acid	0.083 ± 0.6 ^c,d,e,f,g,h^	0.058 ± 1.4 ^c,d,e,f^	0.044 ± 70.0 ^a,b,c^
	Quercetin	0.010 ± 17.2 ^e,f,g,h^	0.105 ± 16.4 ^g,h^	<LOQ ^a^
	Kaempferol	0.062 ± 22.8 ^a,b,c,d,e,f^	0.057 ± 1.8 ^c,d,e,f^	<LOQ ^a^
Maize	-	<LOQ ^a^	<LOQ ^a^	<LOQ ^a^
	(+)-catechin	0.041 ± 37.6 ^a,b,c,d^	0.051 ± ^a,b,c,d,e,f^	0.026 ± 48.7 ^a^
	Epigallocatechin gallate	0.058 ± 68.2 ^a,b,c,d,e^	0.055 ± 20.4 ^b,c,d,e,f^	0.035 ± 31.4 ^a,b^
	*Trans*-ferulic acid	0.080 ± 10.0 ^b,c,d,e,f,g,h^	0.075 ± 7.9 ^e,f,g^	0.050 ± 70.5 ^a,b,c^
	Quercetin	0.113 ± 13.0 ^f,g,h^	0.108 ± 5.7 ^h^	<LOQ ^a^
	Kaempferol	0.069 ± 5.9 ^a,b,c,d,e,f,g^	0.033 ± 23.3 ^a,b,c,d^	<LOQ ^a^

* The results are presented as means (±) relative standard deviation (RSD). Values marked with different letters in columns differ significantly at *p* < 0.05. The limit of quantification (LOQ) < 0.021 g gallic acid/g dry matter. ** Values in bold indicate the highest values within columns. *** The dry matter content of the tested samples was 4.54 ± 0.1% (wheat starch), 5.07 ± 0.2% (rice starch), 4.96 ± 0.4% (potato starch), 4.88 ± 0.2% (maize starch).

**Table 4:** The cytotoxicity was measured by the MTT method [IC50 [µg/mL]].

Sample			Cell Lines	
Starch	Polyphenol	Caco-2	HT29	CCD 841 CoN
Wheat	-	>512 ^c^*	>512 ^c^	>512 ^c^
	(+)-catechin	>512 ^c^	>512 ^c^	>512 ^c^
	Epigallocatechin gallate	>512 ^c^	>512 ^c^	>512 ^c^
	Naringenin	>512 ^c^	>512 ^c^	>512 ^c^
	Quercetin	>512 ^c^	>512 ^c^	>512 ^c^
	Kaempferol	>512 ^c^	>512 ^c^	>512 ^c^
Rice	-	>512 ^c^	>512 ^c^	>512 ^c^
	Epigallocatechin gallate	>512 ^c^	>512 ^c^	>512 ^c^
	Hesperidin	>512 ^c^	>512 ^c^	>512 ^c^
	Naringenin	>512 ^c^	>512 ^c^	>512 ^c^
	*Trans*-ferulic acid	>512 ^c^	>512 ^c^	>512 ^c^
	*p*-coumaric acid	>512 ^c^	>512 ^c^	>512 ^c^
	Quercetin	>512 ^c^	>512 ^c^	>512 ^c^
	Kaempferol	>512 ^c^	>512 ^c^	>512 ^c^
Potato	-	>512 ^c^	>512 ^c^	>512 ^c^
	(+)-catechin	>512 ^c^	>512 ^c^	>512 ^c^
	Epigallocatechin gallate	>512 ^c^	>512 ^c^	>512 ^c^
	Hesperidin	>512 ^c^	>512 ^c^	>512 ^c^
	Naringenin	>512 ^c^	>512 ^c^	>512 ^c^
	*p*-coumaric acid	>512 ^c^	>512 ^c^	>512 ^c^
	Quercetin	275.58 ± 2.1 ^a^	470.51 ± 12.5 ^b^	>512 ^c^
	Kaempferol	>512 ^c^	>512 ^c^	>512 ^c^
Maize	-	>512 ^c^	>512 ^c^	>512 ^c^
	(+)-catechin	>512 ^c^	>512 ^c^	>512 ^c^
	Epigallocatechin gallate	>512 ^c^	>512 ^c^	>512 ^c^
	*Trans*-ferulic acid	>512 ^c^	>512 ^c^	>512 ^c^
	Quercetin	>512 ^c^	>512 ^c^	>512 ^c^
	Kaempferol	>512 ^c^	>512 ^c^	>512 ^c^

* The results are presented as means (±) relative standard deviation (RSD). Values marked with different letters in columns differ significantly at *p* < 0.05. The upper limit of quantification (LOQ)-IC_50_ > 512 µg/mL.

**Table 5:** The samples showing the best results in terms of starch digestibility and estimated glycemic index.

Starch	Polyphenol	Dose of Polyphenol [mg]	Polyphenol Addition Before Starch Pasting	Polyphenol Addition After Starch Pasting
Wheat	-	-	-	-
	(+)-catechin	20	-	+
	Epigallocatechin gallate	20	-	+
	Naringenin	20	-	+
	Quercetin	5	+	-
	Kaempferol	10	+	-
Rice	-	-	-	-
	Epigallocatechin gallate	10	+	-
	Hesperidin	10	+	-
	Naringenin	5	+	-
	*Trans*-ferulic acid	10	+	-
	*p*-coumaric acid	10	+	-
	Quercetin	10	+	-
	Kaempferol	5	+	-
Potato	-	-	-	-
	(+)-catechin	10	-	+
	Epigallocatechin gallate	10	+	-
	Hesperidin	20	-	+
	Naringenin	10	-	+
	*p*-coumaric acid	10	-	+
	Quercetin	20	-	+
	Kaempferol	10	-	+
Maize	-	-	-	-
	(+)-catechin	5	+	-
	Epigallocatechin gallate	20	-	+
	*Trans*-ferulic acid	10	-	+
	Quercetin	5	+	-
	Kaempferol	10	-	+

## Data Availability

The data that support the findings of this study are available from the corresponding authors upon reasonable request.
